# miRCancerdb: a database for correlation analysis between microRNA and gene expression in cancer

**DOI:** 10.1186/s13104-018-3160-9

**Published:** 2018-02-07

**Authors:** Mahmoud Ahmed, Huynh Nguyen, Trang Lai, Deok Ryong Kim

**Affiliations:** Department of Biochemistry and Convergence Medical Sciences and Institute of Health Sciences, Gyeongsang National University School of Medicine, Jinju, 527-27 Republic of Korea

**Keywords:** miRCancerdb, Cancer, MicroRNA, TCGA

## Abstract

**Objectives:**

microRNAs regulate expression of target genes by specifically binding to their transcripts, subsequently leading to translational inhibition or mRNA degradation. Gene regulation by microRNAs has been implicated in a wide range of physiological and pathological conditions. Here, we leverage the use of public-access data and the available genomic annotations to pre-calculate the correlation of the expression of a large number of microRNAs with gene at the mRNA and protein level in the context of cancers.

**Results:**

Expression data of miRNAs, mRNAs and proteins in cancer patients from The Cancer Genome Atlas along with TargetScan miRNAs-target annotations were used to calculate the expression correlations between miRNAs and features (mRNAs/proteins) in a number of cancer studies. We then packed the output of this analysis into a database and made it available through an interactive web application. The miRCancerdb is an easy-to-use database to investigate the microRNAs-dependent regulation of target genes involved in development of cancer.

## Introduction

miRNA (microRNA) are involved, among other functions, in the regulation of gene expression in a wide range of physiological and pathological conditions [1, 2]. Identification of miRNAs and their gene targets is challenging in both bioinformatics and experimental biology. The sequence-based search of gene targets for miRNAs usually yields a high number of false positives [3]. To experimentally confirm these hits, biologist have to go through long lists of potential targets and use laborious laboratory techniques to isolate the meaningful functional associations among them and the miRNAs. The problem is only exacerbated by the diverse expression profiles and functions of miRNAs across tissues and conditions [4].

An attempt to tackle the problem of miRNA target identification while maintaining a reasonable high-throughput is to use a combination of sequence-based search methods and high-throughput expression profiling such as microarrays and RNA sequencing [5, 6]. The process starts by manipulating the expression of a particular miRNA in a certain condition followed by surveying the gene expression by arrays or sequencing. The correlation between the expression of the miRNA and its potential target genes are then calculated [7]. Genes with sequences that match the miRNA seed and correlate strongly in their expression with the miRNA constitute higher chances to be true positives.

Here, we leverage the use of public-access data and the available annotations to pre-calculate the correlation values between the expression of a large number of miRNAs and genes in the context of cancer. We used TCGA (The Cancer Genome Atlas) [8] to obtain miRNA, gene and protein expression profiles in 34 type of cancer and the TargetScan annotation database [9] to calculate the expression correlation for miRNAs and their targets at the mRNA and protein level. We then packed the output of this analysis into a database and made it available in an interactive web application.

## Main text

### Data sources

Expression data of miRNAs, genes and proteins were obtained from TCGA using the R package RTCGA [10]. RTCGA provides the processed counts of these three genetic elements from patient samples using miRNA sequencing, RNA sequencing and Reverse Phase Protein Arrays (RPPA), respectively. Human miRNA target annotations were obtained using the R package targetscan.Hs.eg.db [11]. The miRNA miRBase, gene ENTREZ ID, official gene symbols and manufacturer antibody IDs were used appropriately to connect the data from different assays and sources.

### Analysis pipeline

Expression data from TCGA and target annotations were first processed to a tidy format and different identifiers were translated to common ones. miRNAs and features (mRNA/proteins) were excluded when their expression values were less than 1. Pearson's correlations of miRNA expression matrix and gene expression or protein expression matrices were calculated in each of the TCGA studies (n = 34). The following equation was used as implemented in the R base cor function to calculate the Pearson’s correlation ($\rho$) [12].

$$\begin{aligned} \rho _{X,Y} = \frac{E[(X - \mu _X)(Y - \mu _Y)]}{\sigma _X \sigma _Y} \end{aligned}$$

where, *E* is the expectation, $\mu$ is the mean and $\sigma$ is the standard deviation.

**Fig. 1:**
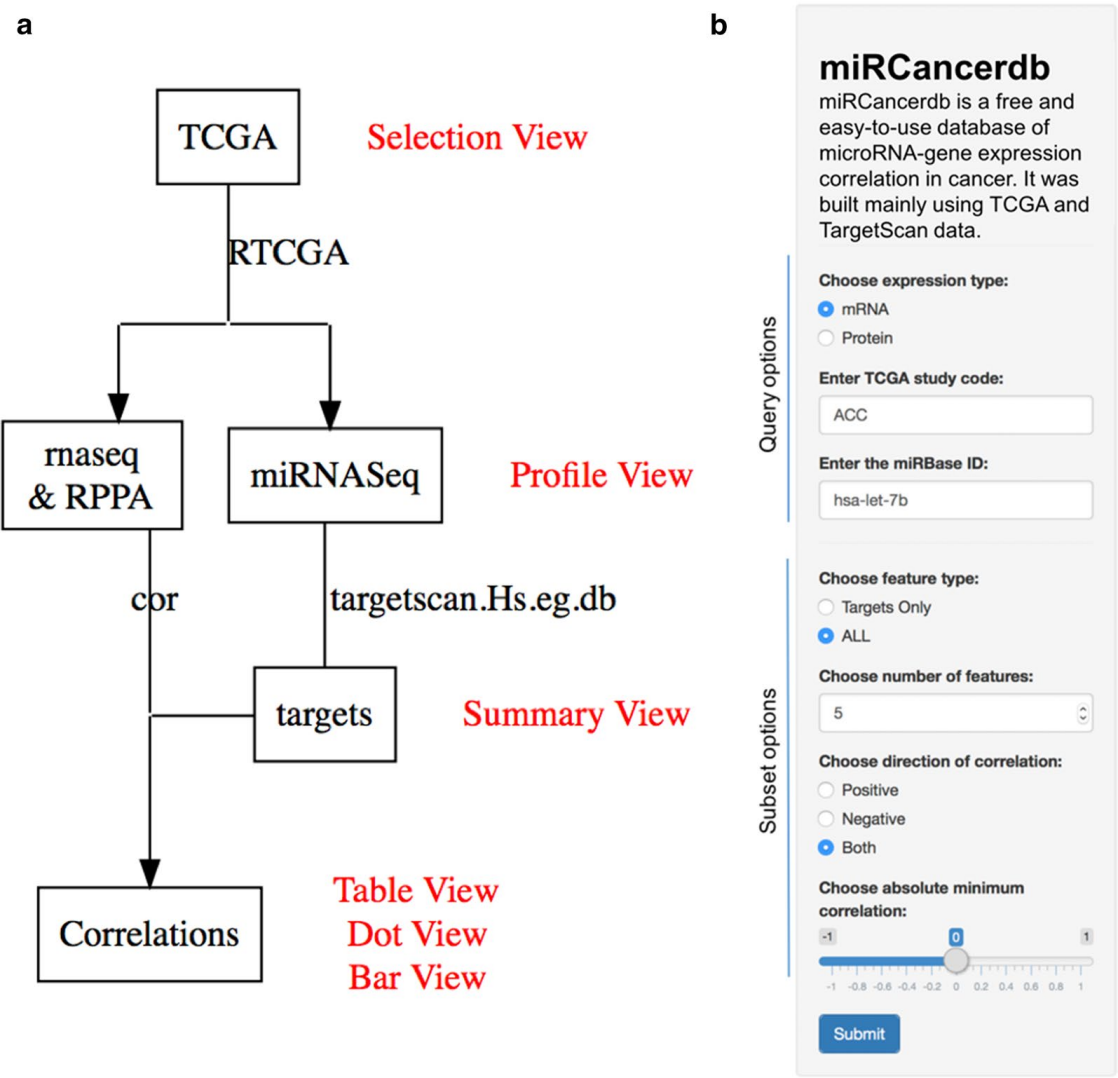
A workflow of the analysis pipeline and miRCancerdb application control panel.** a** The diagram represents the data sources, the analysis pipeline and the representation views. Briefly, expression profiles of three assays; miRNASeq, RNAseq and RPPA from TCGA were obtained using RTCGA package. miRNA gene targets were obtained using the targetscan.Hs.eg.db package. After tidying and filtering the data, Pearsons correlations for each miRNA to features (mRNA/protein) in each of the TCGA studies were calculated. Target features were then identified. Different types of views are available on the web interface to visualize different aspects of queries results. miRNASeq, miRNA sequencing; RNAseq, RNA sequencing; and RPPA, Reverse Phase Protein Array.** b** The panel contains two main areas to enter and customize the query. Query options: to choose the kind of feature expression to base the calculation on, the TCGA study/ies identifier and the miRNAs miRBase IDs. Subset options: to customize the query and limit the results. Options include using feature targets only, limit the results to the top *n* features for each miRNA in each TCGA study, choosing a direction of correlation; positive, negative or both and indicating an absolute minimum correlation to show

Missing values (NA) and correlation values less than 0.1 were omitted to bring the data to a manageable size. Correlation data for each feature in each study were then merged in a single table to build a database file. In addition, a target table of each miRNA and its corresponding targets was added to the database file. Finally, a tidy table of miRNAs expression profiles (counts) in TCGA studies was added to the database file. Figure 1a show the workflow of the analysis and the corresponding output views.

### Database file

The database file is a sqlite3 instance. It contains four tables: *cor_rnaseq* for miRNA and mRNA expression correlations, *cor_rppa* for miRNA and protein expression correlations, *targets* for the miRNA-feature targets and *profiles* for the miRNA expression in TCGA studies.

### Web interface and data presentation

We built an interactive shiny application on top of the database to provide an easy-to-use user interface for miRCancerdb [13]. The web application is available at https://mahshaaban.shinyapps.io/miRCancerdb/. The application main homepage consists of main and control panels. The control panel (Fig. 1b) is where the user can input a search query and customize the results. The results are shown on the main panel in different representations/views (Table 1). In addition, we provide an explanatory text for getting started, how-to and about sections to give an overview and detailed user’s instruction on the website.

**Table 1:** miRCancerdb query results representations/views.

View	Content
Table view	This is a tidy tabular presentation of the search results. The table consists of four columns: *mirna_base*, miRNA miRBase ID; *feature* (mRNA/protein) hits; *study*, TCGA study identifier; and *cor*, calculated correlation
Dot view	This is a point graph representation of the search results. Columns represent each query miRNA in each TCGA study and rows represent feature (mRNA/protein) hits. Each point is scaled by the correlation value and colored by the direction of the correlation; positive or negative. This is a bar graph representation of the search results. Panels represents each query miRNA in each TCGA study. On the x-axis are the feature (mRNA/protein) hits and on the y-axis are the correlation values
Bar view	This is a bar graph representation of the search results. Panels represents each query miRNA in each TCGA study. On the x-axis are the feature (mRNA/protein) hits and on the y-axis are the correlation values
Profile view	This is a point graph representation of the expression profiles of query miRNAs. Each panel represent a TCGA study. On the x-axis are the query miRNAs and on the y-axis are the log count transformation values. Points are jittered on the x-axis for better visibility
Summary view	This is a summary report about the query miRNAs and TCGA studies. It includes three sections. First, pie charts showing the percentage of positive and negative correlated features (mRNA/protein) with each query miRNA in each TCGA study. Second, a table with the total number of targetscan known targets for each miRNA. Finally, density plots showing the distribution of the correlation values of all and target features (mRNA/protein). Each panel represents a TCGA study and curves are colored by the corresponding miRNAs
Selection view	A tool to show the available TCGA studies and miRNAs

**Fig. 2:**
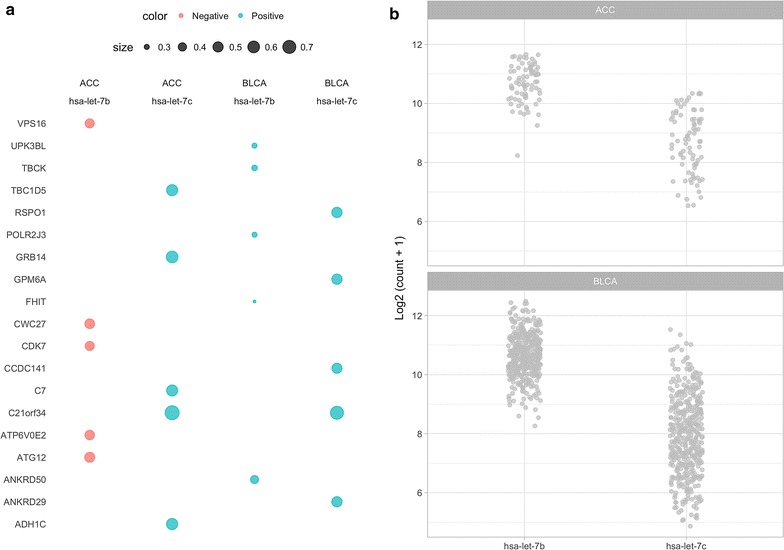
Dot and profile views from a miRCancerdb query use case. A use case of miRCancerdb database to query the miRNAs; has-let-7b and has-let-7c in ACC and BLCA canter studies.** a** A dot view of the top 5 hits of the expression correlation between the miRNAs and genes. Points are scaled by the correlation value and colored by the direction of the correlation; positive (green) or negative (red).** b** A profile view of the expression (log count) of the miRNAs in TCGA studies

### Building and using the database locally

The source files to build and use miRCancerdb locally are available on https://github.com/MahShaaban/miRCancerdb. It consists of R scripts to build the database file and launch an interactive browser application locally. The commands to build the application are wrapped in a makefile that makes it easy to trigger the local build with only a single command.

To do that, download the github repository, navigate to the miRCancerdb directory and run make.
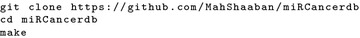


To only run the browser application after the initial build, use the make section, run the R script from the command-line or use within Rstudio.
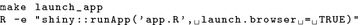


More details about building and using the database locally is available at the database github repository.

### Use case

To illustrate querying the database using the web interactive application, we provide an example of a simple query. Here, we used the query options in the control panel to enter the TCGA identifiers for adrenocortical carcinoma (ACC) and bladder urothelial carcinoma (BLCA) studies, and the miRBase IDs for has-let-7b and has-let-7c. All other options are set to the defaults. Figure 2 show the outputs from the miRCancerdb views.

miRCancerdb provides multiple views to output and represent the results of each query. In this case we show the results from only two. The dot and the profile views. Figure 2a shows the output of the previous query in a dot graph where the queried miRNA on the x-axis and the correlated genes on the y-axis, the figures are faceted by the queried studies. Each dot represent a recorded correlation with the size corresponding to its value and the color to the direction (positive or negative). Figure 2b is a point graph with the miRNAs on the x-axis and the log count in each study on the y-axis. Together, the dot and profile views show the correlated genes, the magnitude, direction of the correlation and the distribution of the count of each miRNA in each of the queried studies. To reproduce the exact figures while using the database locally, one needs to build the database first as described above then run the following code in an R session.

We start by loading the required libraries.
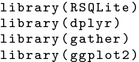


Then, we connect to the database SQLite file and filter the table *cor_rnaseq* to get the correlations of the query of interest.
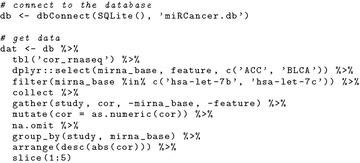


Plot the results as a dot plot (Fig. 2a).
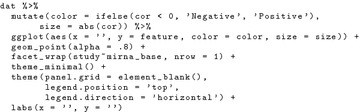


Query the table *profiles* to get the expression of the query of interest.
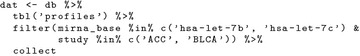


Plot the results as a profile view (Fig. 2b).
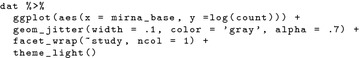


### Utility and discussion

Identification of the regulatory elements (transcription factors and miRNAs) at complex pathological conditions like cancer is a very important step to understand their pathophysiological mechanisms [1, 4]. These regulatory elements often work as a complex network and drive many processes such as cancer growth, differentiation and metastasis [2]. Cistrome Cancer is a database that provides information about transcription factors correlation and regulatory potentials to gene expression in cancer [14]. It is based on integrative analysis of TCGA expression and public access ChIP-seq (Chromatin Immuno-precipitation Sequencing) data. To our knowledge, no tools have been developed to address the similar involvement on miRNAs. miRCancerdb provides pre-calculated expression correlations between miRNAs and mRNAs/proteins in cancers. Its based on an integrative analysis of TCGA public access data and TargetScan annotations.

miRCancerdb can be deployed in a wide variety of research questions. Biologists interested in studying certain miRNAs can use it for their initial surveys. Mainly, the database provides calculated correlations values of these elements to either gene or protein expression in a large number of cancer studies. In addition, it is possible to integrate the available sequence-based annotations to limit the number of highly correlated genes to predicted targets. Also, those who are interested in larger questions can query the database to find out all miRNAs involved in certain types of cancers and compare them to each other. This can be used to discover and compare regulation patterns in neoplastic conditions.

The database file was built with several points in mind. First, we filtered out the less useful information to bring the file size to a manageable size. Second, the file is a stand-alone database which can be accessed programmatically using any SQLite client. Finally, the source code for building the database and launching the browser application is open sourced. This, in one hand leverages the reproducibility and makes updating the database very easy when needed. On the other, the scripts can be used to build the database on local machines and integrate it in different analysis pipelines.

To our knowledge, no available public database provide a data-driven correlation/co-expression of miRNA and genes in cancer. We developed an R package called cRegulome (unpublished) that interfaces the miRCancerdb programmatically from R environment. In addition to the miRNA correlations, the package provide access to transcription factor-gene correlations from the cistrome project [14]. miRCancer is another cancer association database of miRNA based on text mining the the literature [15].

## Limitations

In this kind of integrative analysis based on public access data, we are limited by the available data and annotations. The current miRNA annotations are far from complete. The plan is to keep up with updates from the two major data and annotation sources, namely the TCGA and the TargetScan projects. That is through rebuilding the database with each future updates of the RTCGA and targetscan.Hs.eg.db Bioconductor packages.

In the current version of the database, we treated each TCGA study as one entity. Another plausible way is to consider the cancer heterogeneity and individual variations. This is to stratify the analysis with histological and/or clinical information about the samples. However, this would complicate the pipeline and increase the database size dramatically, so we might address this issue in a later version.

In addition, improving the user experience on the web application and providing graphics that can handle bigger queries is a priority. Finally, providing a similar analysis in the context of human tissues and cell lines using public access data rather than cancer is necessary. This would make a good tool to discover patterns of regulation in many physiological conditions and provides comparison set to these patterns in cancers.

## Abbreviations

ACC: adrenocortical carcinoma
BLCA: bladder urothelial carcinoma
ChIP-seq: chromatin immuno-precipitation (ChIP) sequencing
miRNA: microRNA
RPPA: reverse phase protein arrays
TCGA: The Cancer Genome Atlas

## References

[1] Hayes J, Peruzzi PP, Lawler S (2014). MicroRNAs in cancer: biomarkers, functions and therapy. Trends Mol Med.

[2] He L, Hannon GJ (2004). Correction: MicroRNAs: small RNAs with a big role in gene regulation. Nat Rev Genet.

[3] Thomson DW, Bracken CP, Goodall GJ (2011). Experimental strategies for microRNA target identification. Nucleic Acids Res.

[4] Lu J, Getz G, Miska EA, Alvarez-Saavedra E, Lamb J, Peck D, Sweet-Cordero A, Ebert BL, Mak RH, Ferrando AA, Downing JR, Jacks T, Horvitz HR, Golub TR (2005). MicroRNA expression profiles classify human cancers. Nature.

[5] Lim LP, Lau NC, Garrett-Engele P, Grimson A, Schelter JM, Castle J, Bartel DP, Linsley PS, Johnson JM (2005). Microarray analysis shows that some microRNAs downregulate large numbers of target mRNAs. Nature.

[6] Grimson A, Farh KKH, Johnston WK, Garrett-Engele P, Lim LP, Bartel DP (2007). MicroRNA targeting specificity in mammals: determinants beyond seed pairing. Mol Cell.

[7] Karginov FV, Conaco C, Xuan Z, Schmidt BH, Parker JS, Mandel G, Hannon GJ (2007). A biochemical approach to identifying microRNA targets. Proc Natl Acad Sci.

[8] Hudson TJ, Anderson W, Artez A, Barker AD, Bell C, Bernabé RR, Bhan MK, Calvo F, Eerola I, Gerhard DS, Guttmacher A, Guyer M, Hemsley FM, Jennings JL, Kerr D, Klatt P, Kolar P, Kusada J, Lane DP, Laplace F, Youyong L, Nettekoven G, Ozenberger B, Peterson J, Rao TS, Remacle J, Schafer AJ, Shibata T, Stratton MR, Vockley JG, Watanabe K, Yang H, Yuen MM, Knoppers BM, Bobrow M, Cambon-Thomsen A, Dressler LG, Dyke SO, Joly Y, Kato K, Kennedy KL, Nicolás P, Parker MJ, Rial-Sebbag E, Romeo-Casabona CM, Shaw KM, Wallace S, Wiesner GL, Zeps N, Lichter P, Biankin AV, Chabannon C, Chin L, Clément B, de Alava E, Degos F, Ferguson ML, Geary P, Hayes DN, Hudson TJ, Johns AL, Kasprzyk A, Nakagawa H, Penny R, Piris MA, Sarin R, Scarpa A, Shibata T, van de Vijver M, Futreal PA, Aburatani H, Bayés M, Botwell DD, Campbell PJ, Estivill X, Gerhard DS, Grimmond SM, Gut I, Hirst M, López-Otín C, Majumder P, Marra M, McPherson JD, Nakagawa H, Ning Z, Puente XS, Ruan Y, Shibata T, Stratton MR, Stunnenberg HG, Swerdlow H, Velculescu VE, Wilson RK, Xue HH, Yang L, Spellman PT, Bader GD, Boutros PC, Campbell PJ, Flicek P, Getz G, Guigó R, Guo G, Haussler D, Heath S, Hubbard TJ, Jiang T, Jones SM, Li Q, López-Bigas N, Luo R, Muthuswamy L, Ouellette BF, Pearson JV, Puente XS, Quesada V, Raphael BJ, Sander C, Shibata T, Speed TP, Stein LD, Stuart JM, Teague JW, Totoki Y, Tsunoda T, Valencia A, Wheeler DA, Wu H, Zhao S, Zhou G, Stein LD, Guigó R, Hubbard TJ, Joly Y, Jones SM, Kasprzyk A, Lathrop M, López-Bigas N, Ouellette BF, Spellman PT, Teague JW, Thomas G, Valencia A, Yoshida T, Kennedy KL, Axton M, Dyke SO, Futreal PA, Gerhard DS, Gunter C, Guyer M, Hudson TJ, McPherson JD, Miller LJ, Ozenberger B, Shaw KM, Kasprzyk A, Stein LD, Zhang J, Haider SA, Wang J, Yung CK, Cros A, Liang Y, Gnaneshan S, Guberman J, Hsu J, Bobrow M, Chalmers DR, Hasel KW, Joly Y, Kaan TS, Kennedy KL, Knoppers BM, Lowrance WW, Masui T, Nicolás P, Rial-Sebbag E, Rodriguez LL, Vergely C, Yoshida T, Grimmond SM, Biankin AV, Bowtell DD, Cloonan N, deFazio A, Eshleman JR, Etemadmoghadam D, Gardiner BB, Kench JG, Scarpa A, Sutherland RL, Tempero MA, Waddell NJ, Wilson PJ, McPherson JD, Gallinger S, Tsao MS, Shaw PA, Petersen GM, Mukhopadhyay D, Chin L, DePinho RA, Thayer S, Muthuswamy L, Shazand K, Beck T, Sam M, Timms L, Ballin V, Lu Y, Ji J, Zhang X, Chen F, Hu X, Zhou G, Yang Q, Tian G, Zhang L, Xing X, Li X, Zhu Z, Yu Y, Yu J, Yang H, Lathrop M, Tost J, Brennan P, Holcatova I, Zaridze D, Brazma A, Egevard L, Prokhortchouk E, Banks RE, Uhlén M, Cambon-Thomsen A, Viksna J, Ponten F, Skryabin K, Stratton MR, Futreal PA, Birney E, Borg A, Børresen-Dale AL, Caldas C, Foekens JA, Martin S, Reis-Filho JS, Richardson AL, Sotiriou C, Stunnenberg HG, Thoms G, van de Vijver M, Calvo F, Birnbaum D, Blanche H, Boucher P, Boyault S, Chabannon C, Gut I, Masson-Jacquemier JD, Lathrop M, Pauporté I, Pivot X, Vincent-Salomon A, Tabone E, Theillet C, Thomas G, Tost J, Treilleux I, Calvo F, Bioulac-Sage P, Clément B, Decaens T, Degos F, Franco D, Gut I, Gut M, Heath S, Lathrop M, Samuel D, Thomas G, Zucman-Rossi J, Lichter P, Eils R, Brors B, Korbel JO, Korshunov A, Landgraf P, Lehrach H, Pfister S, Radlwimmer B, Reifenberger G, Taylor MD, von Kalle C, Majumder PP, Sarin R, Rao TS, Bhan MK, Scarpa A, Pederzoli P, Lawlor RA, Delledonne M, Bardelli A, Biankin AV, Grimmond SM, Gress T, Klimstra D, Zamboni G, Shibata T, Nakamura Y, Nakagawa H, Kusada J, Tsunoda T, Miyano S, Aburatani H, Kato K, Fujimoto A, Yoshida T, Campo E, López-Otín C, Estivill X, Guigó R, de Sanjosé S, Piris MA, Montserrat E, González-Díaz M, Puente XS, Jares P, Valencia A, Himmelbauer H, Quesada V, Bea S, Stratton MR, Futreal PA, Campbell PJ, Vincent-Salomon A, Richardson AL, Reis-Filho JS, van de Vijver M, Thomas G, Masson-Jacquemier JD, Aparicio S, Borg A, Børresen-Dale AL, Caldas C, Foekens JA, Stunnenberg HG, van’t Veer L, Easton DF, Spellman PT, Barker AD, Chin L, International Cancer Genome Consortium (2010). International network of cancer genome projects. Nature..

[9] Agarwal V, Bell GW, Nam JW, Bartel DP (2015). Predicting effective microRNA target sites in mammalian mRNAs. Elife.

[10] Kosinski M, Biecek,P. RTCGA: the cancer genome atlas data integration. 2016. https://rtcga.github.io/RTCGA. Accessed 8 Dec 2017.

[11] Csardi G. Targetscan.Hs.eg.db: TargetScan miRNA target predictions for human. 2013.

[12] R Development Core Team. R Internals. Vienna, Austria. 2015. doi: 3-900051-14-3. https://cran.r-project.org/doc/manuals/r-release/R-ints.html. Accessed 8 Dec 2017.

[13] Chang W, Cheng J, Allaire J, Xie Y, McPherson J. shiny: Web Application Framework for R. 2016. https://cran.r-project.org/package=shiny. Accessed 8 Dec 2017.

[14] Liu T, Ortiz JA, Taing L, Meyer CA, Lee B, Zhang Y, Shin H, Wong SS, Ma J, Lei Y, Pape UJ, Poidinger M, Chen Y, Yeung K, Brown M, Turpaz Y, Liu XS (2011). Cistrome: an integrative platform for transcriptional regulation studies. Genome Biol.

[15] Xie B, Ding Q, Han H, Wu D (2013). miRCancer: a microRNA-cancer association database constructed by text mining on literature. Bioinformatics.

